# Copper-Based Silica Nanotubes as Novel Catalysts for the Total Oxidation of Toluene

**DOI:** 10.3390/nano13152202

**Published:** 2023-07-28

**Authors:** Victor Deboos, Carla Calabrese, Jean-Marc Giraudon, Rino Morent, Nathalie De Geyter, Leonarda Francesca Liotta, Jean-François Lamonier

**Affiliations:** 1 Unité de Catalyse et Chimie du Solide (UCCS), Université de Lille, CNRS, Centrale Lille, Université Artois, UMR 8181, 59000 Lille, France; victor.deboos@univ-lille.fr (V.D.); jean-marc.giraudon@univ-lille.fr (J.-M.G.); 2 Research Unit Plasma Technology (RUPT), Department of Applied Physics, Faculty of Engineering and Architecture, Ghent University, 9000 Ghent, Belgium; rino.morent@ugent.be (R.M.); nathalie.degeyter@ugent.be (N.D.G.); 3 Institute for the Study of Nanostructured Materials (ISMN)-CNR, Via Ugo La Malfa, 153, 90146 Palermo, Italy; carla.calabrese@ismn.cnr.it

**Keywords:** silica nanotubes, copper oxide, toluene total oxidation, reducibility

## Abstract

Cu (10 wt%) materials on silica nanotubes were prepared via two different synthetic approaches, co-synthesis and wetness impregnation on preformed SiO_2_ nanotubes, both as dried or calcined materials, with Cu(NO_3_)2.5H_2_O as a material precursor. The obtained silica and the Cu samples, after calcination at 550 °C for 5 h, were characterized by several techniques, such as TEM, N_2_ physisorption, XRD, Raman, H_2_-TPR and XPS, and tested for toluene oxidation in the 20–450 °C temperature range. A reference sample, Cu(10 wt%) over commercial silica, was also prepared. The copper-based silica nanotubes exhibited the best performances with respect to toluene oxidation. The Cu-based catalyst using dried silica nanotubes has the lowest T_50_ (306 °C), the temperature required for 50% toluene conversion, compared with a T_50_ of 345 °C obtained for the reference catalyst. The excellent catalytic properties of this catalyst were ascribed to the presence of easy copper (II) species finely dispersed (crystallite size of 13 nm) on the surface of silica nanotubes. The present data underlined the impact of the synthetic method on the catalyst properties and oxidation activity.

## 1. Introduction

Volatile organic compounds (VOCs) have very harmful effects on health, and some VOCs are carcinogenic, mutagenic and toxic for reproduction [1]. Moreover, by degrading in the atmosphere through a series of photochemical reactions under the effect of sunlight and heat, they can cause the formation or accumulation in the environment of harmful compounds, such as ozone [2]. VOC treatment is therefore necessary in many industrial sectors, such as paint, automotive, chemical and printing industries. Catalytic oxidation allows VOC removal in accordance with regulatory requirements and with a low energy cost. Indeed, the catalytic process leads to a total oxidation of VOCs at a lower temperature than that encountered in thermal oxidation. Transition metal oxide catalysts are developed as a cheaper alternative to noble metal-based catalysts for the total oxidation of VOCs [3]. Among the different transition metal oxides, Cu, Co and Mn oxides are the most active for the total oxidation of different VOCs. Their catalytic activity is influenced by several factors, such as the preparation method and the nature and morphology of the support, with the dispersion and reducibility of the metal species on the support surface being the most significant [4,5,6,7,8,9].

In the case of supported copper oxides, the formation of highly dispersed copper oxide species on the support can be achieved, but for relatively limited copper loadings. Thus, Kim et al. [10] found that it is difficult to introduce more than 5 wt% copper in order to prepare a catalyst with highly dispersed copper species over alumina. Indeed, in the case of copper loading varied from 5 to 15 wt%, the authors showed the toluene conversion decreased with increasing copper loadings. Similarly, Chlala et al. [11] showed that the conversion rate of toluene is highest over Cu (2.5 wt%) supported on hydroxyapatite. The authors showed the formation of larger CuO particles with Cu content increase that are less active in the total oxidation of toluene. The ease of the reducibility of the transition metal oxides is another important factor influencing the catalytic activity in the reaction of VOC oxidation. In this respect, the location of the metal oxide on the support, the nature and morphology of the support (reducible or not reducible oxide) as well as the metal–support mutual interaction can have a significant impact.

Considering silica-supported copper oxides, it was found that the performances of CuO/SiO_2_ catalysts in VOC oxidation were enhanced by tailoring the structure of silica (SBA-15, SBA-16 structures) or modifying the surface of the silica support with promoters such as iron and cerium oxides [12,13,14,15,16]. These promoters can increase the catalytic activity and stability of the active phases by improving their redox properties and also enhancing Lewis acidic properties of the support. Furthermore, the structural and surface properties of the mesoporous silica oxides (SBA-15, SBA-16) play a crucial role in determining the type of copper oxides formed as well as their dispersion and reducibility.

In the search of promising new silica-based solids, with different morphologies, emerged silica nanotubes, prepared by Kruk and co-workers by sol-gel method hydrolyzing TEOS in presence of block copolymer surfactant with long poly(ethylene oxide) blocks, (Pluronic F127, EO_106_PO_70_EO_106_), as templating agent [17].

In this context, Aprile et al. [18,19] disclosed the use of Sn- and Hf-silica nanotubes for glycerol valorization reactions. Recently, Zha et al. [20] claimed a nanocasting method using Co-MOF as a template for the synthesis of Co_3_O_4_ nanoparticles embedded in SiO_2_ nanotubes able to catalyze propane oxidation.

In this work, we broaden the scope of catalytic materials based on hollow silica nanotubes by preparing, for the first time, copper-based silica nanotubes with Cu content (10 wt%) to be used for VOC oxidation. We applied two methods: (i) the post-synthetic approach via the wetness impregnation of preformed SiO_2_ nanotubes with Cu(NO_3_)_2_·2.5H_2_O as a copper precursor; and (ii) the co-synthetic approach by introducing the copper nitrate precursor directly during the silica nanotube synthesis. The obtained samples were tested in the oxidation of toluene, which was chosen as a target VOC molecule. TEM, N_2_ physisorption, XRD, Raman, H_2_-TPR and XPS characterizations underlined the impact of the synthetic method on the properties and catalytic performances. A CuO material dispersed on commercial silica (Aerosil) was also prepared and investigated as reference.

## 2. Results and Discussion

### 2.1. TEM

**Figure 1:**
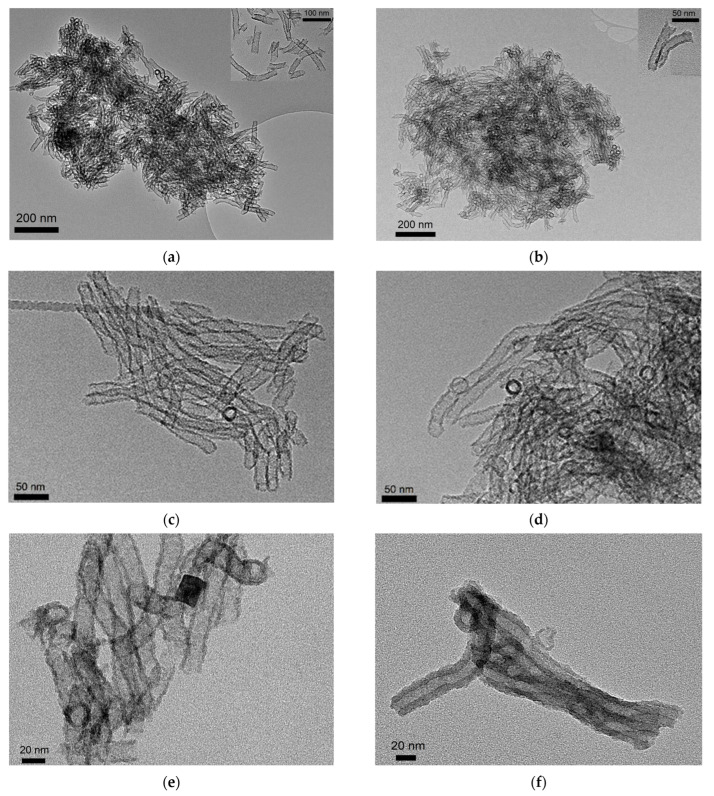
(**a**,**c**) TEM micrographs of SiO_2_-D, (**b**,**d**) TEM micrographs of SiO_2_, (**e**) TEM micrographs of Cu/SiO_2_-D, and (**f**) TEM micrographs of Cu/SiO_2_.

The morphology of the dried (SiO_2_-D) and calcined (SiO_2_) silica supports was examined using transmission electron microscopy (TEM). Figure 1 reports a comparison between the two portions of silica supports. TEM micrographs evidenced that the dried and calcined supports were homogeneously made up of well-defined tubes. The nanotubes were aggregated without any specific order. For the SiO_2_-D sample, the silica nanotubes were already formed, as already shown by some authors [17,21,22]. For the calcined sample, the tubular shape of the silica is reminiscent of the F127 surfactant micelles, which were removed by calcination after the drying step. Silica nanotubes have a length of several hundred nanometers and an external diameter of ~20 nm. The wall thickness can be estimated to ~3 nm. A small quantity of hollow nanospheres were also observed coming from the fragmentation of nanotubes to nanospheres as a result of a budding process, which has already been explained by Loverde et al. [23]. Ultrasonic treatment with ethanol before the TEM analysis showed that the nanotubes are open at both ends (see the two insets in Figure 1a,b.

Copper silica-based materials have also been examined by TEM (Figure 1e,f). No CuO-based nanoparticles were detected in the TEM images. However, while the silica wall thickness of Cu/SiO_2_-D is in the same range as that of the pure support SiO_2_-D (3 nm), that of Cu/SiO_2_ is much higher and can be estimated to ~9 nm (Figure 1f).

### 2.2. N_2_ Physisorption

**Figure 2:**
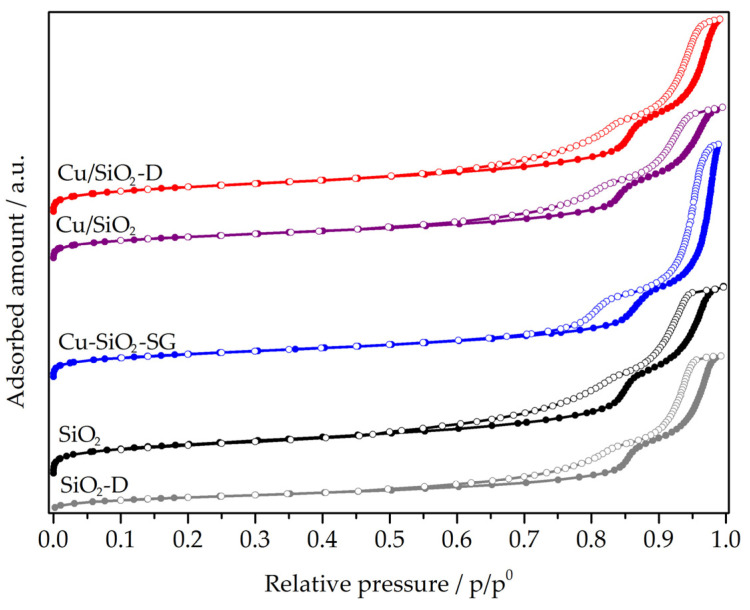
N_2_ adsorption–desorption isotherms of SiO_2_ nanotubes and Cu-supported materials.

**Figure 3:**
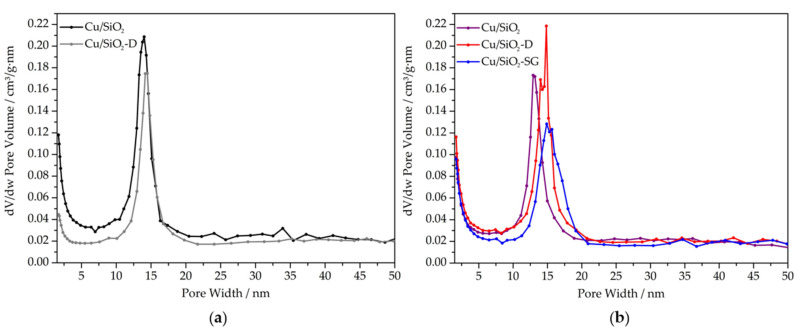
(**a**) Pore size distribution curves of SiO_2_ nanotubes. (**b**) Pore size distribution curves of Cu-supported materials.

The textural properties of the copper–silica nanotubes and pristine supports were addressed by means of nitrogen physisorption measurements. N_2_ adsorption–desorption isotherms and pore size distribution curves of both, silica supports and copper-modified silica nanotubes are displayed in Figure 2 and Figure 3, respectively. All samples exhibit type IV isotherms, with two hysteresis loops assigned to the void space among the nanotubes for the hysteresis between p/p^0^ = 0.90–0.99 and the hollow nanotubes channel at p/p^0^ = 0.65–0.90, as already described for this type of material [24,25].

Compared to the study by Huang et al. [17], which inspired the synthesis of calcined silica nanotubes (SiO_2_), a higher specific surface area (SSA) (1014 m^2^.g^−1^), a similar total pore volume (2.2 cm^3^.g^−1^) and a lower pore size distribution (centered at ~14 nm) are found (Table 1). The pore diameter of ~ 14 nm agrees with the mean values of the inner diameter of the nanotubes (14 nm) estimated by TEM analysis. The BJH method was applied to the adsorption branch of the isotherms, showing a narrow pore size distribution (PSD) centered at 13–15 nm. For dried silica (SiO_2_-D), the SSA is much lower (399 m^2^.g^−1^), in agreement with the presence of the micelles of Pluronic F127 as a structure directing agent inside the nanotubes [17]. The presence of pores with diameters of ~14–15 nm and lower pore volumes (1.9 cm^3^.g^−1^) with respect to the calcined oxide (2.2 cm^3^.g^−1^) confirmed the above hypothesis. This regular pore size distribution of calcined silica nanotubes matched the thermal removal of Pluronic F127 micelles. The pore size distribution remains centered at ~14 nm, a consistent result with the formation of nanotubes in a dried state, according to TEM investigations.

All the copper-based materials exhibit a lower specific surface area in comparison with the calcined support (SiO_2_) (Table 1). This result could be explained by the presence of copper species that may block some channels of nanotubes. Among all the Cu-modified SiO_2_ nanotubes, the Cu/SiO_2_-D material displayed the highest specific surface area (830 m^2^.g^−1^), a result which can be ascribed to the wetness impregnation method applied to just dried SiO_2_ nanotubes. An intermediate specific surface area of 732 m^2^.g^−1^ was recorded when the copper precursor was inserted during the sol-gel synthesis of silica nanotubes. Conversely, the wetness impregnation of calcined SiO_2_ nanotubes produced the lowest specific surface area, equal to 719 m^2^.g^−1^, with respect to SiO_2_ nanotubes subjected to two calcination treatments (before and after copper impregnation). Pore size distribution around 13–15 nm was confirmed for all the Cu silica nanotube catalysts (Figure 3).

For the Cu/SiO_2_-Aerosil material prepared on commercial silica, the specific surface area is much lower (39 m^2^.g^−1^), as is the pore volume (0.1/cm^3^.g^−1^) (Table 1).

### 2.3. XRD

**Figure 4:**
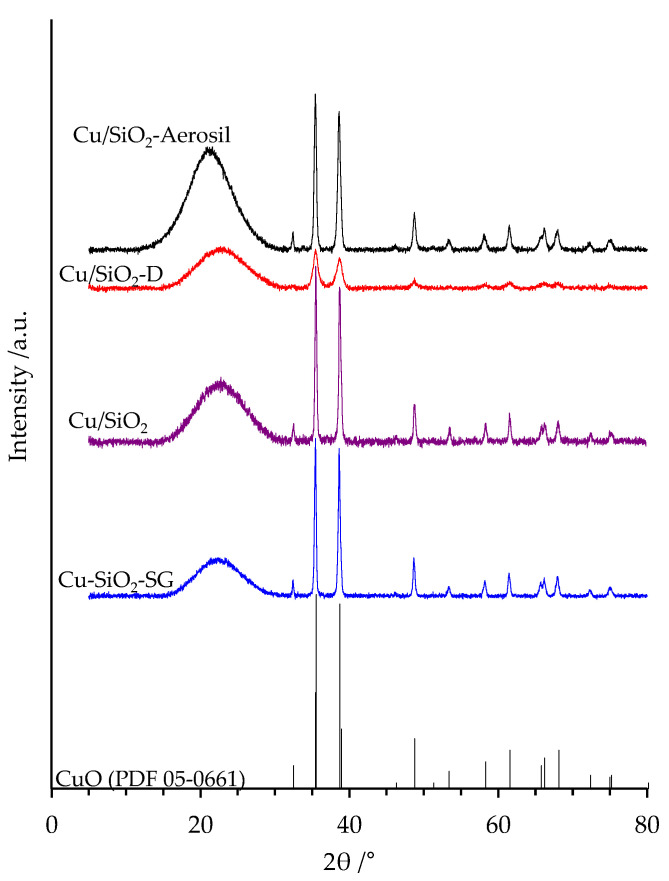
XRD patterns of copper silica-based materials and CuO from PDF #05-0661.

XRD diffractograms of copper-based samples are presented in Figure 4. All samples showed a broad peak centered around 23°, which was already observed for silica nanotubes [26,27,28] and can be ascribed to the presence of SiO_2_ in its amorphous phase. The diffraction peaks at 35.4°, 35.5°, 38.7°, 38.9° and 48.8° correspond, respectively, to the (002), (−111), (111), (200) and (−202) diffraction planes of the CuO tenorite phase (PDF #05-0661). The average crystallite sizes of copper oxide, calculated from the Scherrer equation using the peak at 2θ = 48.8°, are listed in Table 1. The highest crystallite sizes of 43–46 nm are obtained for the samples prepared by wetness impregnation of copper nitrate trihydrate over commercial silica, calcined silica nanotubes and the material prepared by co-synthesis through the sol-gel method. In contrast, when dried silica nanotubes are used for the wetness impregnation of copper nitrate, a lower crystallite size of 13 nm for CuO is obtained. This value fits well with the 14 nm inner diameter of the hollow nanotubes. This finding strongly suggests a good diffusion of copper species inside the hollow silica nanotubes in the course of the hydrothermal treatment. It is likely that the presence of Pluronic F127 in the as-synthesized silica facilitates such behavior, in accordance with different previous studies [29,30]. This result agrees with the highest SSA of the final Cu/SiO_2_-D material (Table 1).

### 2.4. Raman

**Figure 5:**
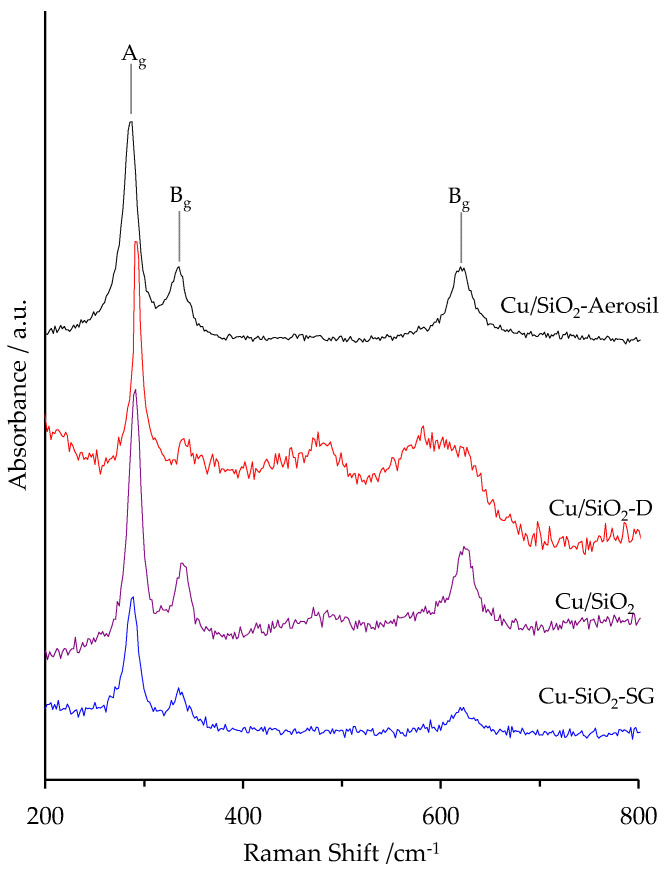
Raman spectra of copper silica-based materials.

Raman spectroscopy experiments were carried out to further investigate the structural features of the prepared samples, as Raman spectroscopy is a very sensitive technique for determining the phase composition of transition metal oxides. The Raman spectra of copper-based particles supported on silica are shown in Figure 5. The monoclinic structure of CuO presents three zone-center optical phonon modes A_g_ and 2B_g_, which are Raman active. For the Cu/SiO_2_-Aerosil sample, three Raman lines attributed to CuO [31] are found at around 284 (A_g_ mode), 337 and 616 cm^−1^ (B_g_ modes) (Figure 5). Those lines are also observed for Cu/SiO_2_-D, Cu/SiO_2_ and Cu-SiO_2_-SG, but with a higher Raman shift. The supplementary broad lines located at around 485 cm^−1^ and 585 cm^−1^ for the Cu/SiO_2_-D and Cu/SiO_2_ samples can be attributed to the silica support, since the same features are observed for pure SiO_2_ (not shown).

### 2.5. H_2_-TPR

H_2_-TPR measurements were carried out in order to investigate the reducibility of the copper species in the silica-supported copper materials prepared by different methods. It is well established that the reduction temperature of CuO varies with the size of the CuO particles and the interaction of these species with the support [32].

**Figure 6:**
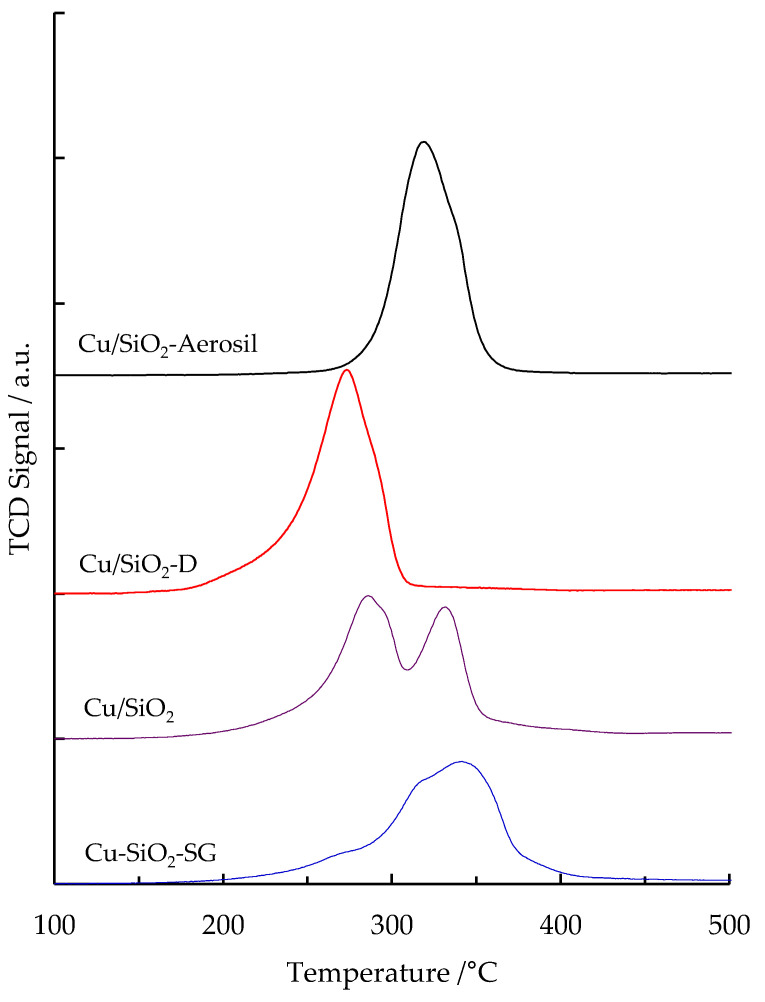
H_2_-TPR profiles of copper silica-based materials.

H_2_-TPR profiles of silica-supported copper materials are shown in Figure 6 and experimental H_2_ consumption is listed in Table 2, where the analytical Cu loading, determined by ICP-OES analysis, is also reported. All samples showed an experimental value H_2_/Cu ratio close to one, which is in agreement with the reduction of Cu^2+^ species into metallic Cu during the H_2_-TPR experiments. For the reference Cu/SiO_2_-Aerosil sample, the TPR profile showed a main peak around 320 °C, with a shoulder at 340 °C. Such profiles have already been observed for many Cu/SiO_2_ samples and can be explained by the presence of two kinds of copper species interacting weakly or strongly with the silica surface [33]. Similarly, the Cu/SiO_2_-D sample presented the same H_2_-TPR profile (Figure 6). However, it is worth noting that the copper species reduction took place at much lower temperatures (maximum of ~265 °C), since the whole profile is shifted by almost 60 °C compared to the profile obtained for Cu/SiO_2_-Aerosil. This result could be related to the lowest size of CuO crystallites evidenced by XRD, which suggests that CuO nanoparticles are well dispersed and in low interaction with the silica support in the Cu/SiO_2_-D sample. The H_2_-TPR profiles are quite different for the Cu/SiO_2_ and Cu-SiO_2_-SG samples (Figure 6). Two distinct H_2_ consumptions at 290 °C and 335 °C are registered for the Cu/SiO_2_ sample. Likewise, Kong et al. [34] observed two peaks for the reduction of copper species, at 248 and 328 °C, for a copper material supported on MCM-41. The authors attributed the low-temperature reduction peak to copper species highly dispersed on the surface of the support and the high-temperature reduction to CuO nano-aggregates. P.M. Cuesta Zapata et al. [35] have shown via DRUV-vis experiments that the preparation of Cu-MCM-41 using the sol-gel method could enable the formation of SiO-[Cu–O–Cu]_n_-CuOSi, SiO–Cu–O–Cu–OSi and SiO–Cu–OSi species, but also small CuO aggregates. Therefore, the complex tail with four different maxima observed for the Cu-SiO_2_-SG sample may be the fingerprint of the reduction of such copper species at different temperatures [36,37].

### 2.6. XPS

**Figure 7:**
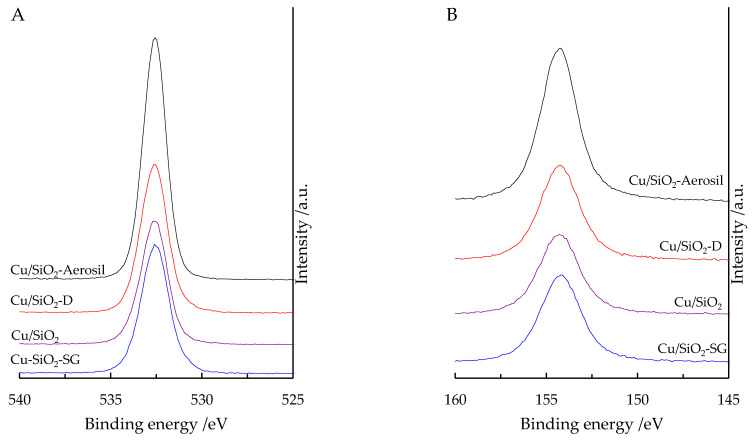
High-resolution O1s (**A**) and Si2s (**B**) XPS spectra of Cu-based materials.

XPS was performed to evaluate the chemical oxidation state of the elements and their relative surface abundance. For all the samples, binding energies (BE) of electrons coming from orbitals O1s (532.6 eV) and Si2s (154.3 eV) (Figure 7) as well as the atomic ratio O/Si calculated from the intensity of these two photopeaks (Table 2) agree with the presence of SiO_2_ [38]. A value slightly higher than two for the XPS O/Si atomic ratio (Table 2) can be explained by the presence of additional oxygen bounded to copper species in the O1s region.

**Figure 8:**
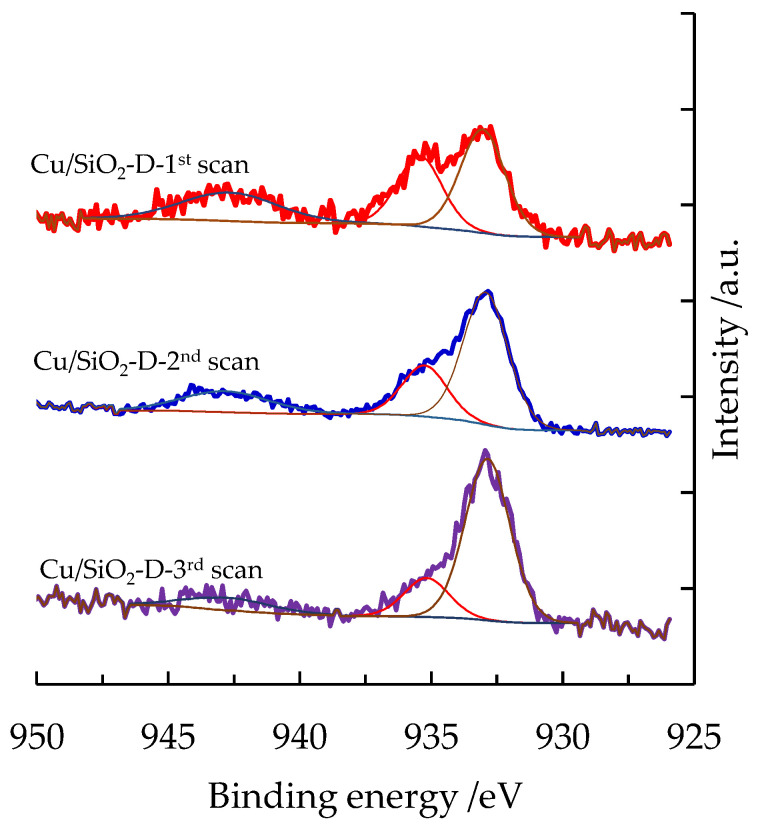
Evolution of the high-resolution Cu 2p_3/2_ signal during XPS analysis for Cu/SiO_2_-D material (experimental and fitted peaks).

The Cu2p region has been carefully examined. The low content of copper detected at the surface of the Cu/SiO_2_-Aerosil sample, pointing to the presence of aggregated CuO particles due to the very low surface area of the material, did not allow for a precise analysis of the Cu2p signal. The appearance of the well-known shake-up satellite is found in the Cu2p_3/2_ spectra and indicates the presence of Cu(II) species. A pronounced decrease in intensity of the shake-up satellite and the presence in the main peak of Cu2p_3/2_ of two contributions at 935.3 eV and 933 eV are observed following three different analyses of the 2p region of copper. Figure 8 shows, for the Cu/SiO_2_-D sample, the evolution of the high-resolution Cu 2p_3/2_ signal during XPS analysis. BE at 935.3 and 933 eV are consistent with the values of 935.4 and 932.8 eV found for well-dispersed CuO and Cu_2_O over the silica support, respectively [39]. These observations suggest that Cu(II) species are reduced during the XPS analyses. The degree of reduction of Cu(II) can be estimated by calculating the ratio of the intensity of the satellite peak to that of the main peak (I_sat_/I_main_) (Table 2). Indeed, the main emission peak contains both Cu(II) and Cu(I) contributions, while the satellite intensity is entirely from Cu(II) [40]. It must be pointed out that the reduction process under a vacuum is much more marked for the Cu/SiO_2_-D, since a reduction level of 66% during the XPS analysis is observed between the first and the third analyses for this sample compared to 22% and 53% for the Cu/SiO_2_ and Cu-SiO_2_-SG samples. These results for reducibility under a vacuum corroborate those obtained by H_2_-TPR, which had highlighted the reducibility at a lower temperature for the Cu/SiO_2_-D sample.

For all Cu-based silica nanotube samples, the XPS Cu/Si atomic ratio values are much lower than the bulk Cu/Si atomic ratios, calculated by ICP-OES analysis and ranging around 0.08–0.1. This finding is consistent with the high surface area and porosity typical of silica nanotubes, which can hide Cu species dispersed inside the pores and depend on the nature of specific material.

The very low value detected in the Cu-SiO_2_-SG sample agrees with the preparation method that involves the burial of some copper within the support. Moreover, it is likely that the co-synthetic approach did not provide a uniform distribution of the copper species in the bulk silica, consistent with the formation of CuO particles whose average crystallite size was estimated by XRD to be 46 nm. Among the impregnated samples, Cu/SiO_2_-D stands out with a higher-value Cu/Si atomic ratio compared to Cu/SiO_2_. This result suggests the presence of a high quantity of well-dispersed CuO species in the Cu/SiO_2_-D sample, and also agrees with CuO crystallite sizes three times smaller, as identified by XRD.

### 2.7. Catalytic Test

**Figure 9:**
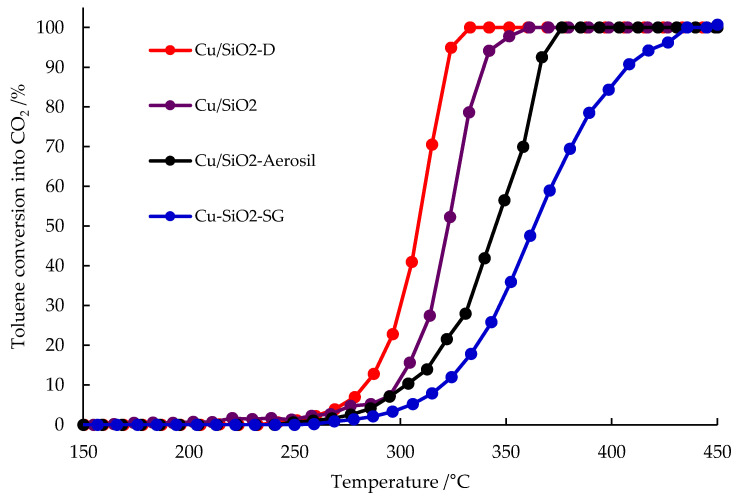
Toluene conversion to CO_2_ over copper silica-based catalysts. Reaction conditions: 200 mg catalyst, 1000 ppm toluene in air, 100 mL.min^−1^ total flow.

The four copper-containing silica catalysts were tested for the total oxidation of toluene. Toluene oxidation leads to the production of CO_2_ as the only carbon product, regardless of the copper silica-based catalyst used. Figure 8 shows the evolution of toluene conversion into CO_2_ as a function of temperature and the nature of the catalyst. T_X_ (the temperature at which X% of toluene has been converted to CO_2_) and the specific rate calculated at 287 °C (*r*_287_) are given in Table 3. Based on these data and the relative position of the light-off curves (Figure 9), catalyst activity can be ranked as follows: Cu/SiO_2_-D > Cu/SiO_2_ > Cu/SiO_2_-Aerosil > Cu-SiO_2_-SG.

**Figure 10:**
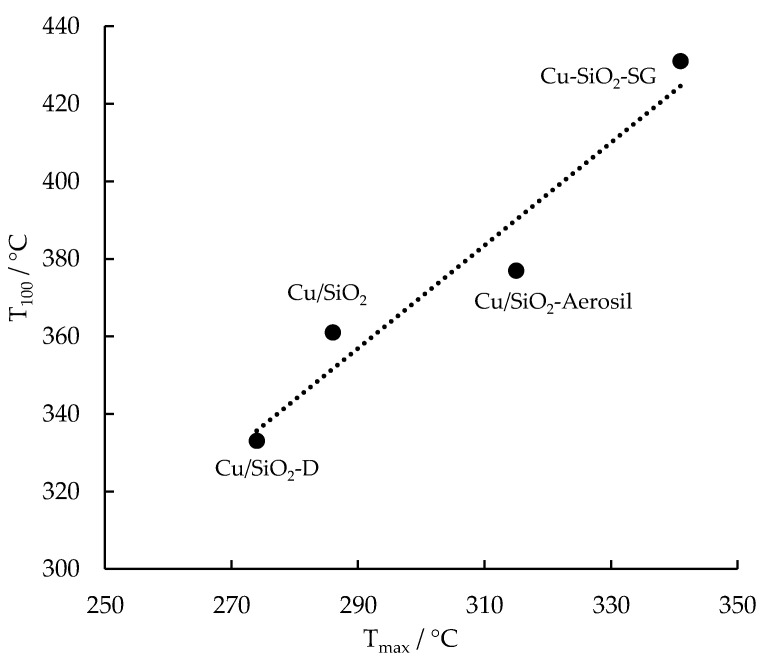
Correlation between the reducibility of copper-based catalysts and their activity in toluene oxidation. T_max_: temperature at which H_2_ consumption is at its maximum; T_100_: required temperature for 100% conversion of toluene to CO_2_.

Despite the higher dispersion of copper oxide particles at the surface for Cu-SiO_2_-SG compared to Cu/SiO_2_-Aerosil, lower activity in the toluene oxidation was observed in the case of Cu-SiO_2_-SG. This result can be connected to the presence of hard copper (II) species in Cu-SiO_2_-SG, highlighted by the H_2_-TPR experiment. The key role of reducibility in VOC oxidation activity based on a Mars–van Krevelen mechanism has already been underlined for metal-oxide-based catalysts [41,42,43], and the relationship between the reducibility of these materials and catalytic activity has been proposed in many papers [44,45,46,47,48]. This correlation is found in Figure 10, showing the dependence of the temperature required for complete oxidation of toluene into CO_2_ (T_100_ from Figure 9) as a function of the temperature at which H_2_ consumption is at its maximum (T_max_—Table 2). The higher the reducibility of copper oxide supported on silica, the greater its toluene oxidation ability. The best catalytic activity obtained in the presence of Cu/SiO_2_-D or Cu/SiO_2_ in terms of the total oxidation of toluene can be related to high copper oxide dispersion at the surface of the silica nanotubes, as demonstrated by XPS analysis. Such copper oxides are easily reducible, as shown via H_2_-TPR analyses. Cu/SiO_2_-D is characterized by the presence of highly reducible copper oxide nanoparticles that allow for the oxidation of toluene at lower temperatures.

The toluene conversion data presented herein show that the Cu/SiO_2_-D and Cu/SiO_2_ catalysts exhibited the best catalytic performances in terms of full oxidation of toluene and compare very well with copper-based catalysts supported over silica oxides tested in similar conditions (see Table 4).

## 3. Materials and Methods

### 3.1. Synthesis of Copper Silica-Based Materials

Silica nanotubes were prepared by the sol-gel method according to the procedure used by Kruk and co-workers [17]. A total of 5.0 g of Pluronic F127 (EO_106_PO_70_EO_106_, Sigma-Aldrich, Milan, Italy) was dissolved in 300 mL of 2M HCl at 11 °C and stirred for 1 h at 250 rpm. Then a solution of TEOS (98%, 0.065 mol, 14.8 mL) in 15 mL of toluene was added dropwise to the reaction mixture and stirred at 250 rpm for 24 h at 11 °C. The mixture was transferred to a polypropylene bottle (with a volume of 500 mL) and kept at 100 °C for 24 h. The as-synthesized material (gel) was then filtered, washed with deionized water (3 L) and dried at 65 °C for 24 h, resulting in a white powder. A portion was used for copper impregnation as is (SiO_2_-D), and another portion was calcined under static air at 550 °C for 5 h (heating ramp 2 °C/min) to remove the surfactant template (SiO_2_). Indeed, TGA analysis of SiO_2_-D under flowing air has shown that Pluronic F127 oxidation takes place at around 400 °C.

Cu 10 wt% was impregnated (copper precursor: Cu(NO_3_)_2_.2.5H_2_O, supplied by Sigma-Aldrich) using the wetness impregnation method over dried and calcined silica nanotubes. After the impregnation step, the samples were dried at 100 °C for 19 h. Finally, all the materials were calcined under static air at 550 °C for 5 h, with a heating rate of 2 °C.min^−1^. In summary, two catalysts were then obtained and labeled Cu/SiO_2_-D—for copper supported on dried silica followed by a calcination step—and Cu/SiO_2_—for copper supported on calcined silica followed by a second calcination step.

A co-synthetic approach, introducing the copper nitrate precursor directly during silica nanotube synthesis, was also used. In a glass container, 5 g of Pluronic F127 (EO_106_PO_70_EO_106,_ Aldrich) was dissolved in 300 mL of 2 M HCl at 11 °C and stirred for 1 h (250 rpm). Then, a solution of tetraethyl orthosilicate (TEOS 98%, 0.065 mol, 14.8 mL) in toluene (15 mL) was added dropwise. Cu(NO_3_)_2_·2.5H_2_O (98%, 6.81·10^−3^ mol, 1.62 g) was dissolved in 2.5 mL of distilled water to be added dropwise to the reaction mixture. The reaction mixture was stirred at 250 rpm for 24 h at 11 °C. After this time, the reaction mixture was transferred to a PP bottle and hydrothermally treated for 24 h at 100 °C. The overall reaction mixture was transferred to a glass crystallizer to be dried at 100 °C for 19 h. The obtained solid (9.95 g) was ground into an agate mortar and then calcined at 550 °C under static air for 5 h, with a heating ramp of 2 °C.min^−1^. The sample prepared by this method was labeled Cu-SiO_2_-SG.

For comparison purposes, a copper material supported on commercial silica was synthesized. A 10 wt% copper material was prepared by wet impregnation of copper nitrate trihydrate (0.56 g) with commercial silica (Aerosil OX50^®^, Degussa, Essen, Germany) (1.35 g), which was previously calcined at 550 °C for 5 h with a 2 °C.min^−1^ ramp under static air. After the impregnation, the sample was dried for 19 h at 100 °C and finally calcined at 550 °C for 5 h. This sample was labeled Cu/SiO_2_-Aerosil.

### 3.2. Characterization of Copper Silica-Based Materials

Elemental analysis of the samples was carried out by inductively coupled plasma optical emission spectroscopy (ICP-OES) using an Agilent 5100 instrument in axial viewing mode. The materials were pretreated in an acidic solution with aqua regia at 120 °C for complete dissolution.

The TEM experiment was performed on a Thermo Fisher Scientific Tecnai G2-20 microscope operating at 200 kV and equipped with LaB_6_. The study of the morphology was performed in bright field imaging conditions using the parallel illumination mode. The TEM samples were prepared via dry powder deposition on lacey carbon Cu grids (200 mesh). Some TEM samples were prepared via dry powder deposition in ethanol after ultrasonication for 10 min on lacey carbon Cu grids (200 mesh).

Nitrogen adsorption–desorption measurements were carried out at −196 °C using Micromeritics ASAP 2020 equipment. The Brunauer–Emmett–Teller (BET) method was applied to the adsorption curve in the standard pressure range of 0.05–0.3 p/p^0^ [50]. Through analysis of the desorption curves, using the Barrett–Joyner–Halenda (BJH) method with the Kruk–Jaroniec–Sayari (KJS) correction [17], the pore volume and pore size distributions were also obtained. Prior to data collection, each sample (≈200 mg) was outgassed under vacuum at 250 °C for 3 h.

X-ray diffraction (XRD) data were collected using a Bruker D8Advanced AXS diffractometer equipped with a Cu K_α1_ monochromatic radiation source (λ = 1.5418 Å) and operated at 40 kV and 30 mA. X-ray diagrams were recorded within the 5–80° region in 2θ with a 0.02° step size (step time = 1 s). Identification of the crystalline phases was performed using EVA software comparing the registered pattern with the ICDD PDF-4 database cards. The sample was crushed to obtain powder to fill the XRD sample holder and smoothed flat. The crystallite size of copper oxide was determined by the Scherrer equation:

(1) $$D=\frac{k\lambda }{\beta \cos\left(\theta \right)}$$

where *D* is the crystallite size, *k* is a constant dependent on the shape of crystallite (fixed at 0.89 by considering it spherical), *θ* is the Bragg angle and *β* is the corrected full peak width at half-maximum intensity:

(2) $$\beta =\sqrt{{{FWHM}_{exp}}^{2}-{{FWHM}_{LaB_{6}}}^{2}}$$

*FWHM_exp_* is the experimental *FWHM* determined by using Origin software for the studied diffraction peak, and *FWHM_LaB6_* is the FWHM at 2θ = 30° of the LaB_6_ used as reference.

Raman spectra were collected with an XploRA PLUS Raman Microscope (HORIBA Jobin Yvon, Palaiseau, France) equipped with a CCD silicon detector cooled by means of a Peltier device. Raman analysis was conducted using a laser wavelength of 532 nm with an output power of 20 mW, which was reduced by attenuation with a filter of 1% on the sample and a grating of 1200 (750 nm). For all conducted analyses, a 100× std objective was employed, resulting in a laser spot of 0.72 μm. The Raman spectrometer was controlled using the software package LabSpec 6. The presented spectra corresponded to the average of 3 scans, with 600 s acquisition time for each scan. The spatial resolution was about 100 μm. All measurements were taken at room temperature. Preceding the Raman analysis, a calibration step was performed using a silicon wafer characterized by the Si line at ῡ = 520.7 cm^−1^.

The H_2_-Temperature programmed reduction (H_2_-TPR) was carried out using a Micromeritics Autochem II 2920 apparatus. The setup was composed of a thermal conductivity detector (TCD) and a U-shaped quartz microreactor. Experimental conditions were fixed following parameters determined by Monti and Baiker [51]. The correct amount of sample to obtain a value of K and P equal to 100 s and 35 °C, respectively, was pre-treated under argon for 1 h at 150 °C. After cooling under argon, the temperature was increased at a ramp of 15 °C.min^−1^ up to 600 °C, with a flow of 50 mL.min^−1^ of 5% H_2_/Ar gas mixture.

X-ray photoelectron spectroscopy (XPS) was performed on an AXIS Ultra^DLD^ spectrometer (Kratos Analytical, Manchester, UK) with a monochromatic Al Kα X-ray source (hν = 1486.6 eV). High-resolution spectra were collected with a constant pass energy (PE = 20 eV), and the binding energy was calibrated with the peak of Si 2p with a binding energy of 103.3 eV [52]. Quantification and spectral decomposition were processed using CasaXPS software (Version 2.3.17).

### 3.3. Catalytic Performance Evaluation

For each catalytic test, 200 mg of each sample was sieved to have a particle size of 100 μm < dp < 200 μm, placed in a Pyrex reactor between 2 g of SiC (VWR Chemical, 0.5 mm) on each side and activated at 450 °C for 1 h under a flow of air with a ramp of 5 °C.min^−1^. Then, toluene (1000 ppm in air) passed through the reactor, with a total flow rate of 100 mL.min^−1^ at 450 °C for 2 h; the temperature then decreased to room value with a ramp of 0.5 °C.min^−1^. Toluene conversion to CO_2_ (τ_CO2_) was calculated by the formula reported in Equation (3), with *C_out,CO_*_2_ and *C_in,tol_* as the concentration of CO_2_ at the exit and the initial concentration of toluene, respectively.

(3) $$Toluene\;conversion\;into\;{CO}_{2}\left(\%\right)=\frac{C_{out,{CO}_{2}}}{{7\times C}_{in,tol}}\times 100$$

The concentrations were evaluated by GC (7860A Agilent Gas Chromatography, Santa Clara, CA, USA) equipped with a thermal conductivity detector (TCD), a flame ionization detector (FID) and 2 columns: a Restek Shin Carbon ST/Silco HP NOC 80/100 micro-packed column and a capillary column (Cp-Was 52CB25 m, Ø = 0.25 mm) to separate permanent gas and hydrocarbons and aromatic compounds, respectively.

Specific rate at 287 °C (*r*_287_) was calculated using Equation (4), where *F*_0_ is the initial flow rate of toluene (6 × 10^−3^ L.h^−1^), τ_CO2_ is the conversion of toluene into CO_2_, *V_m_* is the molar volume (24.5 L.mol^−1^ in the STP conditions) and *m* is the mass of the catalyst (g) inside the reactor.

(4) $$r_{287}\left(\mu mol.h^{-1}.g^{-1}\right)=\frac{F_{0}\cdot \tau_{{CO}_{2}}}{V_{m}\cdot m}.10^{6}$$

## 4. Conclusions

Novel copper-based silica nanotubes with 10 wt% Cu content were successfully prepared and tested for toluene total oxidation. Two preparation strategies were employed: a post-synthetic method by wetness impregnation of preformed SiO_2_ nanotubes (both dried powder and calcined) with copper nitrate; and a co-synthetic approach via direct introduction of a copper nitrate precursor during silica nanotube synthesis. TEM micrographs confirmed the formation of silica nanotubes with a length of several hundred nanometers and an external diameter of around 20 nm for both dried and calcined silica supports. When using the dried portion of the silica nanotubes for the impregnation of copper species, the corresponding materials (Cu/SiO_2_-D) had outstanding features. The highest-value Cu/Si atomic ratio (0.038) suggested the presence of a high quantity of well-dispersed CuO species, and also agrees with CuO crystallite sizes three times smaller (13 nm), as identified by XRD. The light-off temperature for the total oxidation of toluene was 17 °C lower than the temperature recorded for the sample prepared in the conventional way (using calcined silica nanotubes prior to copper impregnation), resulting in agreement with the reduction of copper species at much lower temperatures for Cu/SiO_2_-D.

## Figures and Tables

**Table 1:** Specific surface area and pore volume of supports and copper silica-based materials and crystallite size of CuO in copper silica-based materials.

	Specific Surface Area /m^2^.g^−1^	Pore Volume /cm^3^.g^−1^	Crystallite Size of CuO ^1^ /nm
Cu/SiO_2_-Aerosil	39	0.1	44
Cu/SiO_2_-D	830	2.3	13
Cu/SiO_2_	719	1.8	43
Cu-SiO_2_-SG	732	2.8	46
SiO_2_	1014	2.2	-
SiO_2_-D	399	1.9	-

^1^ Calculated from the Scherrer equation using the peak at 2θ = 48.8°.

**Table 2:** Data extracted from H_2_-TPR and XPS measurements.

Sample	T_max_ ^1^/°C	H_2_/Cu	Cu/wt% ^2^	O1s/eV ^3^	Si2s/eV ^3^	I_sat_/I_main_ (Cu2p_3/2_) ^3^			Atomic Ratio ^3^	
						1st Analysis	2nd Analysis	3rd Analysis	Cu/Si	O/Si
Cu/SiO_2_-Aerosil	315	0.97	7.9	532.6	154.3	nd *	nd *	nd *	0.003	2.09
Cu/SiO_2_-D	274	0.94	10.1	532.6	154.3	0.35	0.22	0.12	0.038	2.29
Cu/SiO_2_	286	0.88	9.8	532.6	154.3	0.41	0.17	0.22	0.028	2.26
Cu-SiO_2_-SG	341	0.97	8.2	532.6	154.2	0.37	0.18	0.17	0.012	2.22

* not determined. ^1^ Temperature at which H_2_ consumption is at its maximum. ^2^ From ICP-OES analysis. ^3^ From XPS analysis.

**Table 3:** Tx (the temperature at which X% of toluene has been converted to CO_2_) and specific rate *r*_287_ at 287 °C.

Sample	T_20_/°C	T_50_/°C	T_100_/°C	*r*_287_/μmol.h^−1^.g^−1^
Cu/SiO_2_-Aerosil	320	345	377	54
Cu/SiO_2_-D	294	306	333	147
Cu/SiO_2_	308	323	361	65
Cu-SiO_2_-SG	336	363	431	25

**Table 4:** Comparison of the catalytic efficiency of the Cu-based silica nanotubes with available literature data on Cu-based silica.

Catalyst	Reaction Mixture Composition	GHSV/h^−1^	T_100_/°C	Ref.
Cu/SiO_2_-Aerosil Cu/SiO_2_-D Cu/SiO_2_ Cu-SiO_2_-SG	0.2 g 100 mL.min^−1^ 1000 ppm of toluene	26	377 333 361 431	This work
9 wt% Cu/SBA-16 9 wt% Cu/SBA-15	30 mg 30 mL/min^−1^ P_toluene_ = 0.9 kPa	1.2	447 ^a^ 447 ^b^	[13]
5 wt% Cu/SiO_2_	0.1 g 160 mL.min^−1^ 230 ppm of toluene	19	>500	[49]
5 wt% Cu/SiO_2_	0.5 g 60 mL.min^−1^ 900 ppm of toluene	6	>350	[10]

^a^: at T ~ 447 °C, the conversion (%) was close to 87.5%. ^b^: at T > 447 °C, the conversion (%) was >87.5%.

## Data Availability

No new data were created.
